# Case of Sarcoma of the Ileum, Etc.

**Published:** 1893-04

**Authors:** James E. Thompson

**Affiliations:** M. B,. B. S., Lond., Univ., F. R. C. S. Eng.; Prof. of Surgery, Med. Department, Univ., Tex., Galveston


For Daniel's Texas Medical Journal:

### CASE OF SARCOMA OF THE ILEUM—PERFORATION—PERITONITIS—ABDOMINAL SECTION—DEATH IN 10 DAYS.

BY JAMES E. THOMPSON, M. B,. B. S., LOND., UNIV., F. R. C. S. ENG.
[Prof. of Surgery, Med. Department, Univ., Tex., Galveston.]

W. J. W., A FARMER, aged 19, was admitted under my care to the John Sealy Hospital on Oct. 19th, 1891.

He gave a history of two months illness during which time he had suffered from colicky pains in the abdomen. For some time previously, attacks of constipation had alternated with attacks of diarrhœa. About three weeks before admission symptoms became more severe; pain in the hypogastric region became intense, and in a few days a tumor developed in this situation:

Micturition was very frequent and attended with pain, and constipation was obstinate; no vomiting, high fever, especially in the evenings. Weakness and emaciation followed, and the patient was sent down to Galveston.

On admission I found the following: A tumor in the hypogastric region reaching from the symphysis pubis half way to the Umbilicus, and extending symmetrically outwards to the middle of Poupart's ligament. It was semi-resonant on percussion, but quite resonant near the border. It was hard and fairly well defined; the border not ceasing abruptly, but fading gradually into the healthy abdominal wall.

It was excessively tender and the abdominal muscles were firmly contracted over it.

The bladder contained only a small quantity of perfectly healthy wine.

Examination "per rectum" revealed a hard mass in front which could be perfectly palpated between the finger in the rectum and a sound in the bladder.

There was marked œdema of scrotum and both legs. No vomiting. Constipation was absolute; flatus not even passing. The temperature was 101.2° and the pulse 115, weak and compressible. From the physical and symptomatic evidence I concluded that I was dealing with a "localised peritonitis" and that the tumor was a mass of matted intestines, probably enclosing a purulent collection.

I sought in vain for any clue to the cause, such as affections of the vermiform appendix, rectum or bladder.

The following day an abdominal section was performed, the patient being under the influence of chloroform.

A four inch vertical median incision was made of the most prominent part of the tumor. After a careful dissection through the deeper layers of the abdominal wall, the peritoneum was raised with difficulty from a portion of the anterior surface of the tumor, which was then found to consist of coils of intestine glued together by fairly firm adhesions. Some of these adhesions were carefully separated, when, suddenly, a cavity containing pus and fæcal matter was entered. This was promptly washed out and explored.

As far as could be made out it seemed to occupy the whole of the cavity of the true pelvis between the rectum and the bladder. The cause of the extravasation of fæces was searched for, but without result, so the cavity was thoroughly irrigated, and a glass drainage tube inserted. The greater part of the wound was sewn up, and a dressing of carbolised tow and iodoform gauze applied.

The next day the patient was a little better and the temperature was somewhat lower, but the pulse continued to keep its high rate.

Abdominal distension appeared on the 2nd day after the operation, and obstinate vomiting followed in twenty-four hours.

The patient was then fed on nutrient enemata to the end. The day before death the vomit became stercoraceous.

Constipation remained obstinate throughout; enemata of olive oil, castor oil and turpentine were given, but with no effect. The temperature gradually descended to 97.2 on the third day after the operation, then rose again, reaching 102.6 on the seventh day.

Pulse remained quick and weak during the whole period; never being less than 109 per minute and reaching occasionally 148.

Death occurred on the tenth day.

The results of the autopsy were as follows: The coils of Jejunum and Ileum were congested and greatly distended with flatus. On tracing the distended Ileum downwards it suddenly passed into a mass of peritonitic adhesions, situated just above the symphysis; and here the distension ceased, giving the appearance of stricture.

The lower two feet of the Ileum were tangled in a mass of inflammatory adhesions. A portion of this, about three inches in length, was the seat of a new growth of a hard cartilaginous consistence, and a bright rose color which occupied the whole circumference of the walls.

A perforation with ragged shreddy margins, admitting two fingers, and evidently of old standing, was found in the side of the growth, which was situated behind, and above the bladder,--and firmly adherent.

The performation in the growth communicated with the intestinal canal internally, and opened externally into a large abscess cavity which occupied the whole true pelvis. The Cæcum and vermiform appendix were perfectly healthy, and two inches of healthy Ileum intervened between the growth and the Ileo-cæcal valve.

Bladder and rectum were healthy. There were no secondary deposits in the mesenteric glands and liver.

Examination of the growth shewed it to be a pure round celled sarcoma of the small celled type. No normal bowel structure was to be seen in the specimen.

The question of diagnosis in these cases is always difficult, and in many, a strictly accurate diagnosis is often impossible.

I felt quite sure I was dealing with a localised peritonitis, but all attempts to find a cause were in vain.

Perforation of the appendix seemed the most likely, but it was quite impossible to get any certain information on this point.

There were many things against such a diagnosis, such as:

1. The gradual onset of the disease.

2. No history of a previous attack, although this might have been the first manifestation.

3. The position of the tumor; strictly confined to the middle line, and not in the right Iliac fossa.

4. The absence of any symptoms at any time referable to this region, such as pain, tenderness, baggy swelling, etc.

All of these negative signs, however, may in certain cases be valueless, especially the third, as it is no uncommon thing to find the vermiform appendix hanging down over the brim of the pelvis, and, if in such a case perforation occur, the resulting abscess will occupy the cavity of the true pelvis, giving rise to a tumor with symptoms like the foregoing, and associated with a very prominent symptom, viz., frequent painful micturition, owing to the confined bound down bladder being unable to expand and retain a normal quantity of urine.

In the diagnosis of chronic peritonitis with matting together of loops of intestine, I would like to lay stress on the semi-tympanitic note over the swelling; also on the fact that the edge of the swelling fades gradually into the healthy peritoneal area.

Most solid tumors of a non-inflammatory origin shew a clearly marked and easily palpated edge.

In cases like these, abdominal section is certainly to be advised and that too as soon as possible.

Such operations should be conducted with a strict regard to the best interests of the patient. If possible the growth should be excised and a complete enterectomy done. This however, should only be entertained if the patient's strength allows, and the condition of parts is such as to allow of its proper performance. In certain cases (and the above is one) a prolonged search for the perforation should be discountenanced, as by so doing we increase the dangers to the patient, and run a chance of converting a localised, into a generalised peritonitis. However, the conduct of each operation must be left to the discretion of the operator; as in many cases the perforation can be discovered, and if in the ileum stitched up; if in the vermiform appendix, removed.

